# Macular corneal dystrophy in a Chinese family related with novel mutations of *CHST6*

**Published:** 2009-04-06

**Authors:** Xiuhong Dang, Qingguo Zhu, Li Wang, Hong Su, Hui Lin, Nan Zhou, Ting Liang, Zheng Wang, Shangzhi Huang, Qiushi Ren, Yanhua Qi

**Affiliations:** 1 Department of Ophthalmology, the Second Affiliated Hospital of Harbin Medical University, Harbin, Heilongjiang, China; 2 Department of Medical Genetics, Institute of Basic Medical Sciences, Chinese Academy of Medical Sciences, Beijing, China; 3 Laboratory of Ophthalmology & Visual Optics, Institute for Laser Medicine and Bio-Photonics, Department of Biomedical Engineering, College of Life Science and Technology, Shanghai Jiaotong University, Shanghai, China

## Abstract

**Purpose:**

To identify mutations in the carbohydrate sulfotransferase gene (*CHST6*) for a Chinese family with macular corneal dystrophy (MCD) and to investigate the histopathological changes in the affected cornea.

**Methods:**

A corneal button of the proband was obtained by penetrating keratoplasty. The half button and ultrathin sections from the other half button were examined with special stains under a light microscope (LM) and an electron microscope (EM) separately. Genomic DNA was extracted from peripheral blood of 11 family members, and the coding region of *CHST6* was amplified by the polymerase chain reaction (PCR) method. The PCR products were analyzed by direct sequencing and restriction enzyme digestion.

**Results:**

The positive reaction to colloidal iron stain (extracellular blue accumulations in the stroma) was detected under light microscopy. Transmission electron microscopy revealed the enlargement of smooth endoplasmic reticulum and the presence of intracytoplasmic vacuoles. The compound heterozygous mutations, c.892C>T and c.1072T>C, were identified in exon 3 of *CHST6* in three patients. The two transversions resulted in the substitution of a stop codon for glutamine at codon 298 (p.Q298X) and a missense mutation at codon 358, tyrosine to histidine (p.Y358H). The six unaffected family individuals carried alternative heterozygous mutations. These two mutations were not detected in any of the 100 control subjects.

**Conclusions:**

Those novel compound heterozygous mutations were thought to contribute to the loss of CHST6 function, which induced the abnormal metabolism of keratan sulfate (KS) that deposited in the corneal stroma. It could be proved by the observation of a positive stain reaction and the enlarged collagen fibers as well as hyperplastic fibroblasts under microscopes.

## Introduction

Macular corneal dystrophy (MCD; OMIM 217800) is an autosomal recessive inherited disorder characterized by bilateral progressive stromal clouding and central corneal thinning [1]. The onset usually occurs in the first decade of life, starting with a fine superficial stromal haze in the central stroma. Gradually, the opacification extends to the periphery and usually involves the entire cornea, leading to visual impairment. At this time, penetrating keratoplasty is necessary.

Although clinically indistinguishable, MCD has been subdivided into three immunophenotypes I, IA, and II, based on the serum level of sulfated keratan sulfate (KS) measured by enzyme-linked immunosorbent assay (ELISA) and an immunohistochemical evaluation of the corneal tissue. The serum level of KS and the immunohistochemical reaction to the cornea is negative in the patients with MCD type I. The serum level of KS is negative, and the immunohistochemical reaction to the cornea is positive in the patients with type IA whereas both are positive in those patients with type II [2-5].

MCD is histologically characterized by the accumulation of glycosaminoglycans in the stromal lamellae, underneath the epithelium, and within the keratocytes and endothelial cells [3,6]. An abnormality in the metabolism of KS has been implicated in the pathogenesis of MCD.

A gene responsible for MCD types I and II has been linked to chromosome 16q22 [7-9]. Recently, mutations in a new carbohydrate sulfotransferase gene (*CHST6*) encoding corneal glucosamine N-acety-6-sulfotransferase (C-GlcNac-6-ST) have been identified [10]. Lack of activity of this enzyme is thought to result in the production of unsulfated KS and the loss of transparency in the corneas of affected patients. Akama [10] and others [11-16] have described missense mutations, nonsense mutations, and insertions within the coding region in *CHST6* in patients with type I macular corneal dystrophy (differentiation between types I and IA was not performed) whereas MCD type II is associated with deletions and rearrangements in the upstream region of *CHST6* as well as missense mutations [10,16-19]. Despite the variety of mutations [20] reported in both type I and type II patients, all of the identified sequence changes are associated with a similar clinical appearance [10-15].

In this study, we identified novel compound heterozygous mutations within *CHST6* in a Chinese family with MCD and investigated the histopathological changes of the opaque cornea.

## Methods

### Clinical evaluation

**Figure 1:**
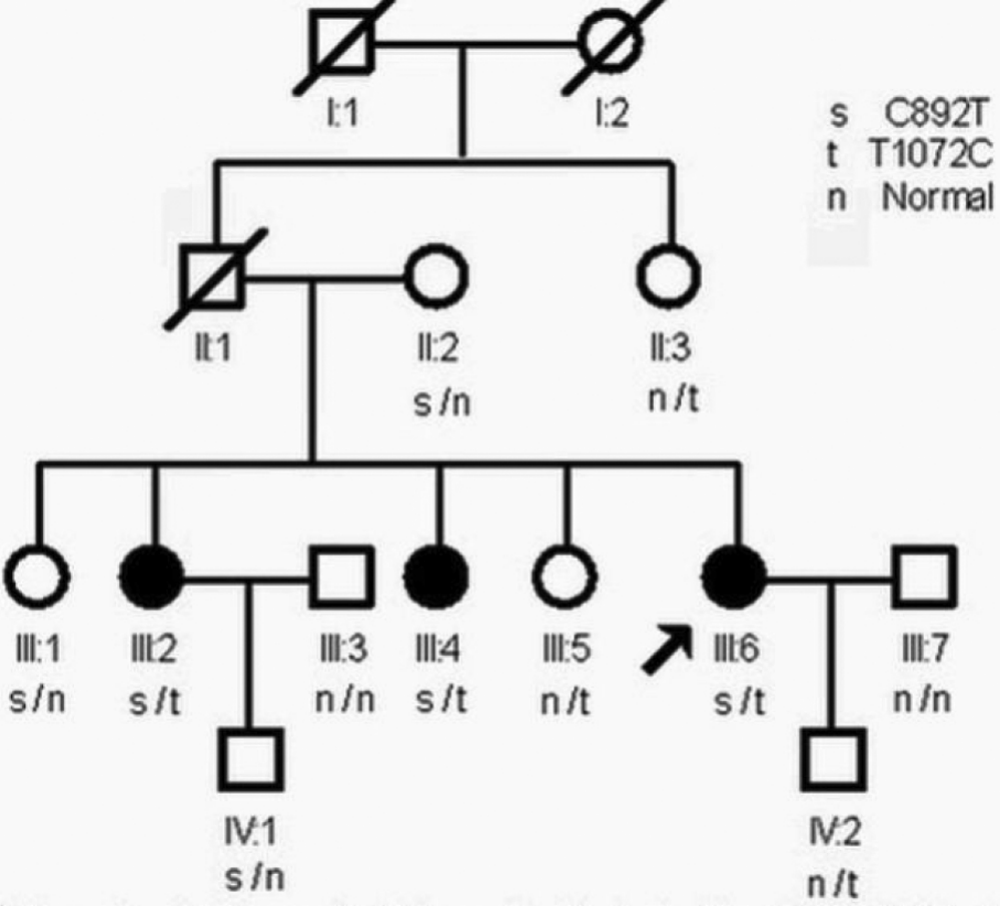
Pedigree of a Chinese family including three individuals affected with MCD. Open symbols indicate clinically unaffected members, filled symbols indicate clinically affected members, and the arrow indicates the proband.

The family was ascertained by the Department of Ophthalmology at the Second Affiliated Hospital of Harbin Medical University (Harbin, China). Informed consent in accordance with the Declaration of Helsinki and the Heilongjiang Institutional Review Board approval was obtained from all participants. The pedigree was a four-generation family with three affected members, six unaffected family individuals, and two spouses (Figure 1). Genomic DNA was extracted from leukocytes of their peripheral blood by standard procedures. Clinical diagnosis of MCD for these patients was based on pedigree structure and clinical features, which include bilateral symmetric stromal cloudiness studded by small, irregular, rounded, gray-white anterior stromal patches. One hundred normal Chinese samples were collected as the control.

### Special stain and ultrastructural features

A half section of the corneal button obtained from the proband by penetrating keratoplasty was fixed in 4% formaldehyde then made into paraffin-embedded samples stained with Hale’s Colloidal iron (Maxim IHC World, Fuzhou, China). After being fixed in 2.5% buffer glutaraldehyde, the other half section was washed with PBS, fixed in 4% osmium tetroxide, dehydrated in the alcohol, and embedded by routine epoxy resin from which ultrathin sections were cut and stained with uranyl acetate and lead citrate. The sections were examined with light microscope (LM) and electron microscope (EM).

### Mutation analysis

The unique exon involving the coding region, exon 3, was amplified using polymerase chain reaction (PCR) with the primers described previously [11]. The cycling program started with an initial denaturing step of 5 min at 95 °C followed by 33 cycles of 94 °C for 30 s, 53–57 °C for 30 s, and 72 °C for 45 s with a final extension step at 72 °C for 10 min. PCR products were purified and directly sequenced on both strands by using an automatic DNA sequencer (ABIPrism 377XL; Applied Biosystems, Foster City, CA).The nucleotide sequences were compared with the published cDNA sequence of *CHST6* (NM_021615).

### Restriction enzyme digestion

Two enzymes, PvuII with recognition site 5′…CAG**ˇ**CTG…3′ and PmacI with recognition site 5′… CAC**ˇ**GTG… 3′, were used for all family individuals to confirm the two heterozygous mutations identified by sequencing c. 892C>T and c.1072T>C, respectively. To exclude the possibility that the mutations were polymorphisms, the PCR products from 100 control subjects were also analyzed by those restriction enzymes. After digestion with PvuII and PmacI, the PCR products were electrophoresed by 6% nondenaturing polyacrylamide gel and visualized by staining with 0.2% AgNO_3_.

## Results

### Clinical findings

**Figure 2:**
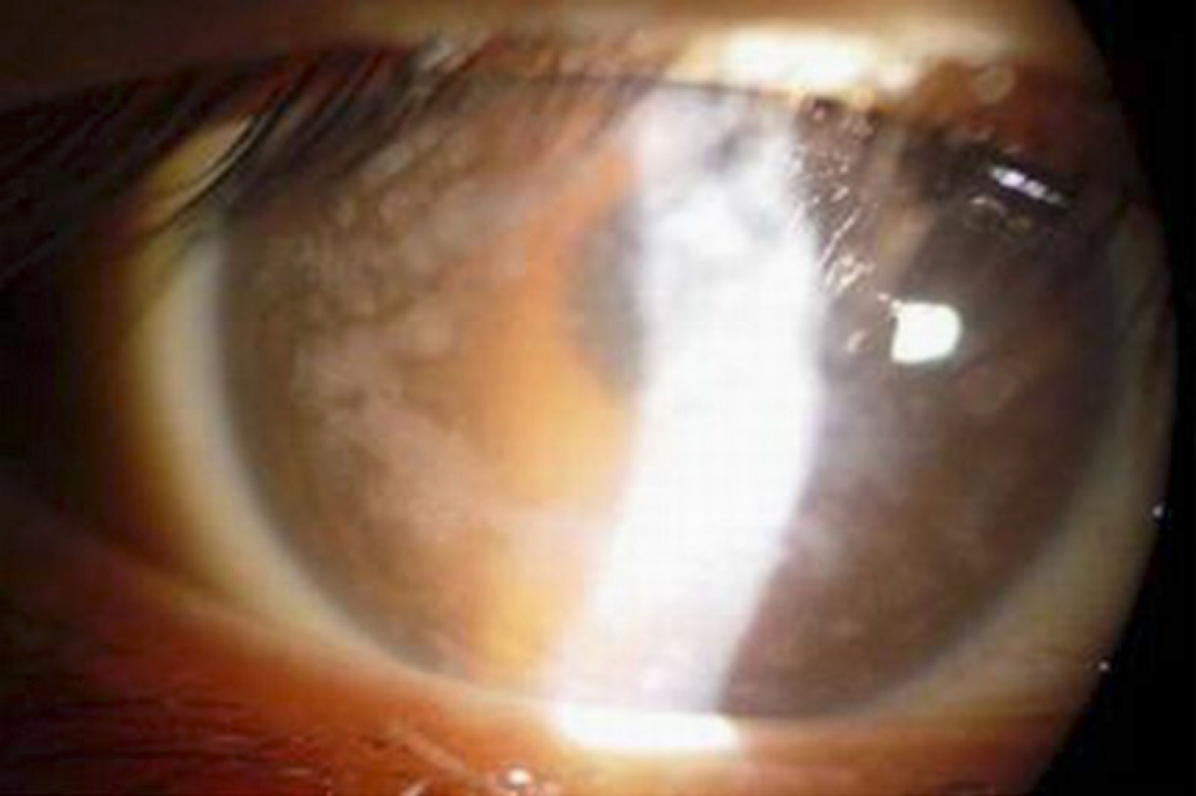
A photograph of the right eye of the proband. It shows stromal cloudiness studded by irregular rounded gray-white opacities in the central and peripheral cornea.

Slit lamp examination of the affected individuals showed that stromal cloudiness were studded by irregular rounded gray-white opacities in both the central and peripheral cornea (Figure 2). The corneal opacities were deep into corneal stroma anterior, the endothelial layer, and Descemet’s membrane.

### Exhibition of histopathology

**Figure 3:**
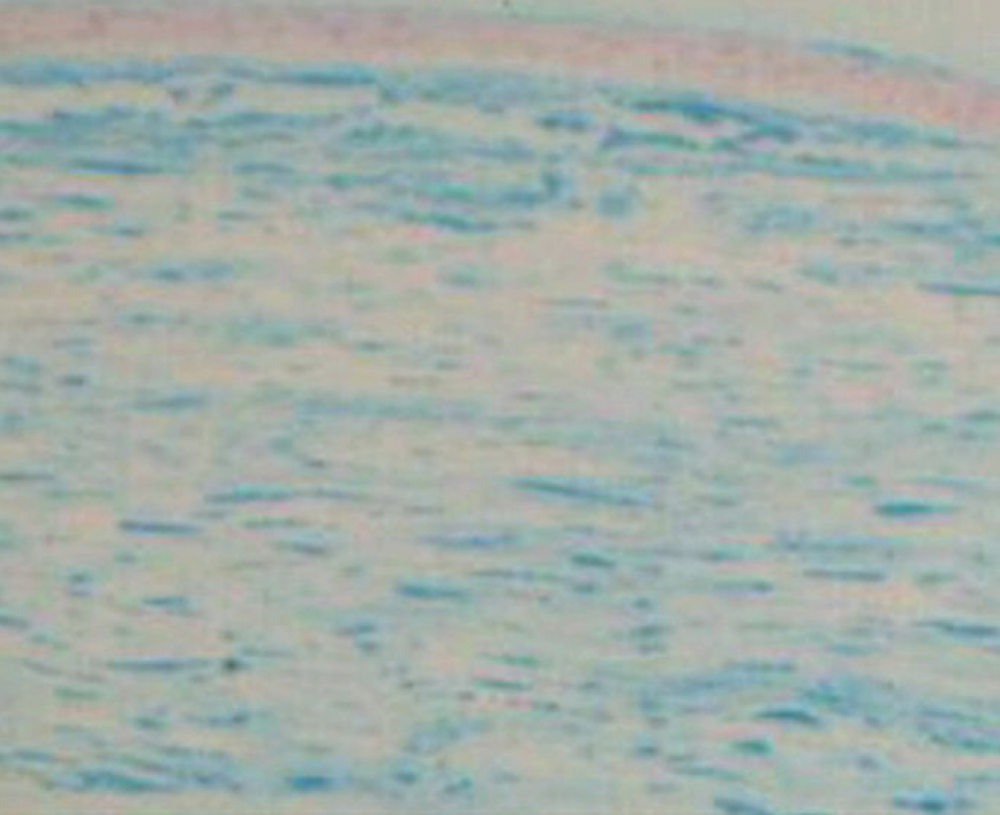
Paraffin section of the proband’s cornea stained with Hale’s colloidal iron. Blue plaque deposits were found in the corneal stroma and under the endothelial layer. These deposits presented a significantly positive reaction to colloidal iron staining.

From light microscopic study, plaque deposits were diffusely found in the corneal stroma and even near the endothelium. They presented significant positive reaction to Hale’s colloidal iron stain, which was indicated by extracellular blue accumulations in the stroma. (Figure 3).

**Figure 4:**
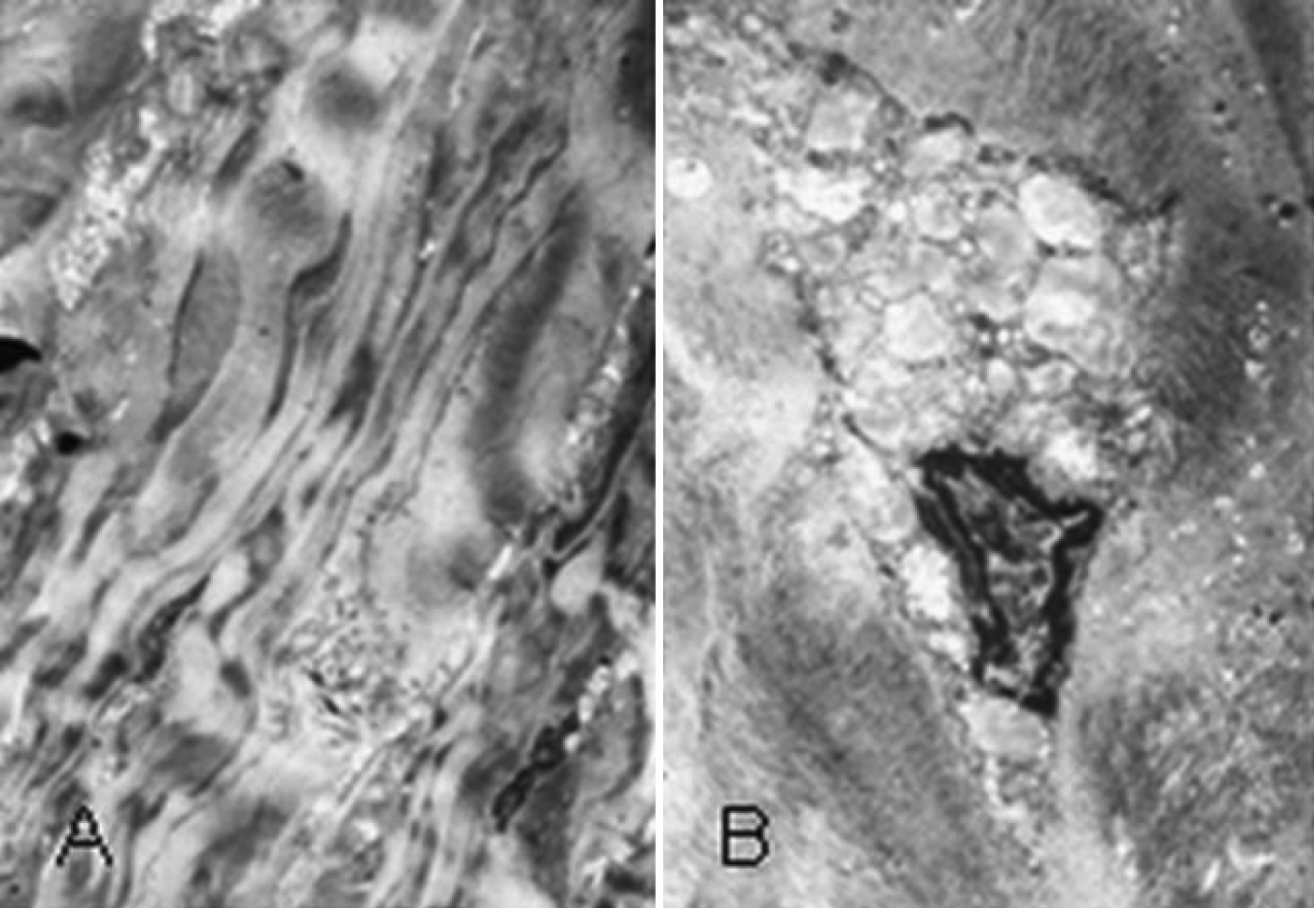
Transmission electron microscopy result of paraffin section of the proband’s cornea stained with Hale's colloidal iron. **A**: The picture shows that the collagen fibers in the corneal stroma are irregular, enlarged, and intensive (1,500X). **B**: The picture shows hyperplastic, active fibroblasts and intracytoplasmic vacuoles in the cornea (5,000X).

Under transmission electron microscopy, it was displayed that the collagen fibers in the corneal stroma were irregular, enlarged, and intensive and the fibroblasts were active and hyperplastic. The enlargement of the smooth endoplasmic reticulum and intracytoplasmic vacuoles appeared within those cells (Figure 4).

### Results of sequencing

**Figure 5:**
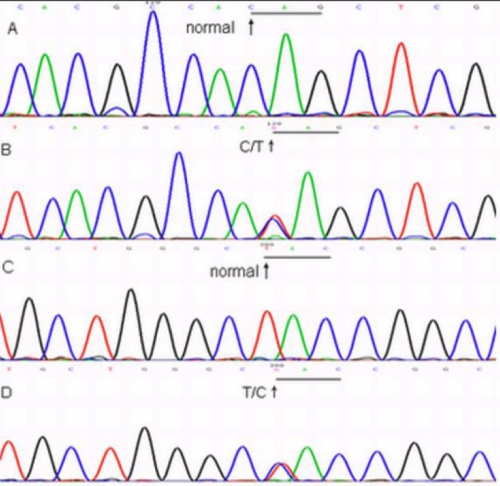
Direct sequencing analysis of the coding region of *CHST6*. **A**: Normal sequence at codon 298. **B**: Sequence of the coding region of *CHST6* in the proband revealed a change of the nucleotide at codon 298 (CAG→TAG). **C**: Normal sequence at codon 358. **D**: Sequence of the coding region of *CHST6* in the proband showed a substitution at codon 358 (TAC→CAC).

*CHST6* is 16.9 kb in length and consists of four exons of which only exon 3 contains the coding region. Two mutations, c. 892C>T and c. 1072T>C, were identified on each allele of exon 3. The first heterozygous change from the maternal allele, a c. 892C>T transition, occurred at the first nucleotide position of codon 298, predicting a change of amino acid glutamine to a stop codon (p.Q298X). The other change on the paternal allele, a c. 1072T>C transition, was identified at the second nucleotide position of codon 358, changing the amino acid from tyrosine to histidine (p.Y358H; Figure 5). Each patient (III:2, III:4, and III:6) in this family was found to have these two mutations within *CHST6*. The unaffected individuals II-2, III:1, and IV-1 were heterozygous with the nonsense change, c. 892C>T, while the other unaffected individuals II-3, III-5, and IV-2 were heterozygous with the missense change, c. 1072T>C.

### Restriction enzyme digestion

PCR products from individuals with c.892C>T transition were digested into 153 bp and 173 bp by restriction enzyme, PvuII, whereas ones without c.892C>T transition only had a 153 bp segment. Sequence with c.1072T>C transition could be divided into 337 bp and 357 bp by PmacI, but those without c.1072T>C transition only had a 357 bp segment. In addition, the results showed that the two heterozygous restriction enzyme segments segregated with all affected members.

**Figure 6:**
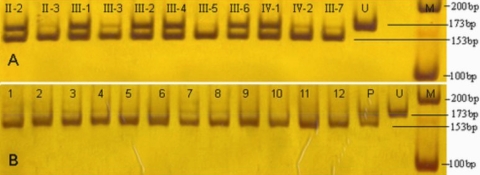
PvuII digestion of PCR products for the c.892C>T transition. PCR products in individuals with the c.892C>T transition were digested into 153 bp and 173 bp and into only 153 bp in individuals without the c.892C>T transition. **A**: Members of the Chinese family with MCD. PCR products from individuals II:2, III:1, III:2, III:4, III:6 and IV:1 with c.892C>T transition were digested into 153bp and 173bp, other members without c.892C>T transition only had a 153bp segment. **B** :Part of 100 control subjects. Control subjects without c.892C>T transition only had a 153bp segment. M indicates DNA ladder, U indicates undigested PCR product, and P indicates one patient with MCD in the Chinese family.

**Figure 7:**
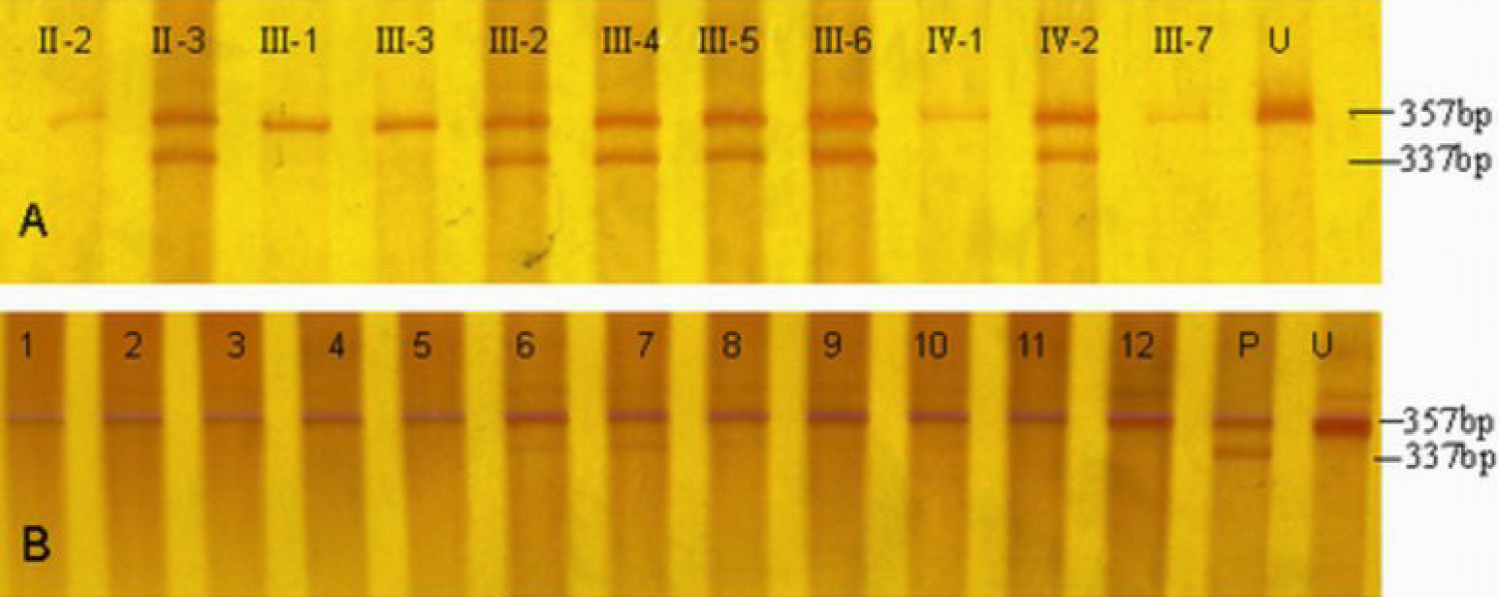
PmacI digestion of PCR products with for the c.1072T>C transition. PCR products in individuals with the c.1072T>C transition were digested into 337 bp and 357 bp and into only 357bp in individuals without the c.1072T>C transition. **A**: Members of the Chinese family with MCD. PCR products from individuals II:3, III:2, III:4, III:5, III:6 and IV:2 with c.1072T>C transition were digested into 337bp and 357bp, other members without c.1072T>C transition only had a 357bp segment. **B**: Part of 100 control subjects. Control subjects without c.1072T>C transition only had a 357bp segment. U indicates undigested PCR product, and P indicates one patient in the Chinese family with MCD.

PCR products from 100 control subjects were analyzed by the same two restriction enzymes, but no mutation segments were found among them (Figure 6, Figure 7).

## Discussion

Macular corneal dystrophy is the least common of the classic stromal dystrophies. This disorder is more common in Iceland, however, representing the most frequent indication for penetrating keratoplasty [21]. In the present study, we collected one pedigree with three patients clinically diagnosed with MCD. Slit lamp examination of the affected individuals showed corneal opacities and stromal haze, which were common characteristic of MCD as described elsewhere [3,22].

Sequence analysis of the *CHST6* coding region revealed compound heterozygous mutations, c.892 C>T and c.1072T >C, co-segregated with the disease in the pedigree, which resulted in a missense mutation (p.Y358H) and an early stop codon (p.Q298X), respectively, and implied MCD as a recessive inherited disorder. Each patient (III:2, III:4, and III:6) diagnosed with MCD in this family were found to have both mutations. Six unaffected blood related members carried either one of the two mutations. None of these nucleotide alterations were detected in the 100 normal control subjects, indicating that these variations were not polymorphisms.

The CHST6 protein is a sulfotransferase, a carbohydrate sulfotransferase of the semi-methyl-D/N-acetyl-galactose/N-acetyl glucosamine-6-O-sulfotransferase family, which can catalyze the phosphorylation of 6-hydroxy-6-O of N-acetyl glucosamine, galactose, and N-acetyl galactosamine. In the normal corneal tissue, keratan sulfate (KS) is an important glycosaminoglycan, which exists as a highly sulfated form and plays a crucial role in the structure and transparency of the cornea. CHST6 can transfer the sulfuric acid group from the 3′-adenosine 5′-phosphate acid (PAPS) to the KS by competing with an endogenous or exogenous substrate [23]. The variations in the coding region of *CHST6* may decrease activity of the enzyme or even make it lost, leading to a low sulfated form or non-sulfated form of KS. Due to the loss of it's soluble property, non-sulfated KS is unable to be completely metabolized, inducing sediment to deposit in the corneal stroma [24].

The nonsense change (p.Q298X) resulted in the premature termination of protein transcription and truncated, dysfunctional protein products. Sultana [11] and Ha [12] also found other nonsense mutations in the gene and thought that the truncated protein was instable and could be degraded rapidly, which was related to the MCD phenotype [14]. The p.Y358H mutation is a new missense mutation that changes tyrosine to histidine, inducing the substitution between a neutral amino acid into a basic amino acid. In 2005, El-Ashry [25] found the p.Y358D mutation (tyrosine to aspartate) in the patients with MCD type I, which changes from a neutral amino acid into an acidic amino acid. The tyrosine at codon 358 is highly conservative in many eukaryotic species such as human, canine, chimpanzee, rat, mouse, and drosophila, using bioinformatic analysis. So we speculated that the Y358H mutation could also affect the protein function [26].

Thus, in our study, KS was observed to deposit in the cornea extensively and deeply, which should be associated with severe abnormalities of the sulfotransferase, leading to little activity and lack of function. As detected by microscopy, plaque accumulations (significant positive reaction to Hale’s colloidal iron stain) diffusely appeared in the corneal stroma and even close to the endothelium. They were likely to be made up of the enlarged and intensive collagen fibers and active, hyperplastic fibroblasts that attempted to ingest the abnormal sediment, which caused the enlargement of smooth endoplasmic reticulum and the presence of intracytoplasmic vacuoles in the cells.

The mutations identified in this Chinese family are completely different from those reported previously in other Asians (Japanese) [10] or whites (British and Icelandic) [11,22]. Together with previous reports, our data indicate significant allelic heterogeneity exists in MCD and the essential role of *CHST6* in the transparency of the cornea. The immunophenotype of MCD has not been performed in our study since we could not test the serum level of KS. Yet no matter the type of MCD, all have indistinguishable clinical characteristic and the same responsible gene. Further studies on these mutations would be helpful to achieve a clearer understanding of the molecular mechanisms of MCD.
